# Formal optimization techniques select hydrogen to decarbonize California

**DOI:** 10.1038/s41598-024-52157-6

**Published:** 2024-01-29

**Authors:** Clinton Thai, Jack Brouwer

**Affiliations:** Advanced Power and Energy Program, University of California, Irvine, CA 92697-3550 USA

**Keywords:** Energy storage, Renewable energy, Energy grids and networks

## Abstract

System planning across economic sectors is becoming increasingly necessary. Building upon existing solutions for deep decarbonization, the inclusion of renewable capacity to meet up to 8 MMT/year hydrogen demand is carried out. An hourly economic dispatch problem modeling the 2050 California electric grid given this hydrogen demand constraint is solved. Hydrogen demand outside of the power generation sector is fixed, but the demand for power generation is endogenously determined. The factor to consider offshore wind capacity, in addition to a conservative and an aggressive hydrogen technology adoption approach, creates four distinct scenarios to evaluate. The difference in results then provides a basis for discussing the costs and benefits associated with using hydrogen to further decarbonize across all sectors. The carbon reduction achieved outside of the power generation sector is 27 MMT despite a slight increase in carbon within the power generation sector. The seasonal storage requirement for hydrogen spans from 72 to 149 TBtu dependent upon the renewable capacity mix. This level of hydrogen demand results in 21% to 41% of total electric load being dedicated to hydrogen production. Battery energy storage has the lowest energy throughput in the middle of the year coinciding with peak electrolyzer operation.

## Introduction

California has the goal of 100% zero emission electricity sales by 2045^1^ with a 50% checkpoint by 2030. While this implies that some portion of sales can include carbon emissions, the state aims to achieve net-zero carbon for power generation, measured at the point of retail sales. Similarly, executive order (EO) B-55-18 aims for the state to be carbon neutral by 2045, largely impacting the transportation sector. SB 129 explicitly dedicates almost 4 billion dollars over three years to zero emission vehicle (ZEV) investments, infrastructure, and clean transportation equity programs^2^. This is largely to bolster the current progress toward meeting the goals established by EO B-48-18^3^, which aims to have 200 hydrogen fueling stations and 250,000 electrical vehicle chargers. Importantly, in California and all other leading decarbonization jurisdictions around the world, emissions reductions in the electric sector have far outpaced those in transportation^4^. The push for decarbonizing the transportation sector provides an opportunity for cross-sectoral evaluation regarding whether the adoption of significant amounts of hydrogen-centric technology is viable and how hydrogen technology may affect the future power generation sector. For clarity, the term “renewable” in this work will generally defer to the definition used in California’s renewable portfolio standard for electricity and derivative fuels otherwise.

Hydrogen as a transportation fuel has the potential to become a major contributor to decarbonization, a driver for increased demand, and is a key variable to consider when analyzing the renewable fuel market. Kluschke and Neumann^5^ consider both the gas and electric grids. They consider hydrogen refueling stations with on-site electrolyzers that can add 72 TWh of electric demand to the 463 TWh per year in the base case. The authors find roughly a 2% total system cost reduction if the refueling stations are planned with electric grid congestion relief in mind. This is because hydrogen can be produced through a process known as electrolysis, where electricity is used to split water to produce oxygen and hydrogen gas, often referred to as power-to-gas in the literature. This process is at the center of renewable studies that are focused on using excess renewable electricity that would otherwise be curtailed to produce hydrogen with essentially zero lifecycle carbon emissions. In addition, electrolyzers can be used as a tool to help manage the dynamics of renewable generation by adding electrical load when necessary. Kluschke and Neumann is a key reference study that simulates a future decarbonized power generation sector with a massively renewable transportation sector fuel demand and evaluating the resulting regional loads and transmission constraints of integrating significant capacity of electrolyzers.

Focused on the environmental aspect, Wang et al.^6^ find using excess renewable electricity for transportation fuels is the most cost-effective way to reduce emissions. Excess electricity sent to energy storage for stationary electric loads have less emissions reduction impacts because current transportation fuels are more greenhouse gas intensive than fuel for power generation. They identify that an approach that minimizes emissions can have significantly differing results from an approach that minimizes costs. This suggests that an optimization that must meet certain emission reduction targets will inherently result in a higher cost solution than if there were no emissions reduction targets. While renewable gas usage for industrial applications provides the greatest total system cost reduction at higher levels of renewable penetration, renewable gas usage in the transportation sector will result in total system cost reduction at lower renewable penetration levels, both use cases enabled by the dispatchable operation of electrolyzers enabling higher renewable deployment. Jentsch et al.^7^ utilize electrolyzers to accommodate an 85% renewable energy supply nodal model of Germany. They identify the lowest cost optimal capacity to deploy found to be between 6 and 12 GW located mostly in the north of Germany to reduce power flows from offshore wind electricity production. Colombo et al.^8^ model a flexible solid-oxide electrolyzer to manage the excess PV generation in a university microgrid setting. This installation enables doubling the amount of solar capacity that can be installed on the campus and the hydrogen produced from excess electricity is used in an on-site gas turbine (GT) reducing 16% of CO_2_ emissions from power generation.

While studies like these from Colombo et al. are important to highlight the benefit and motivation of deploying these novel systems, long-term planning, and outlook studies regarding the bulk system, both electric and gas, are also key in providing guidelines for social expectations and continual financial and policy support.

A comprehensive white paper roadmap for California by Reed et al.^9^ is available which addresses the supply and demand of hydrogen as well as the physical siting of the production plants through to 2050. On the electric side, Colbertaldo et al.^10^ evaluate what 100% renewable energy supply with different mixes of solar, wind, electrolyzer, and fuel cell amounts in California would be necessary, but their work does not consider other sectors outside of power generation. Nonetheless, this is a key work in establishing the magnitude of renewable capacity required to achieve deep decarbonization goals. Between these two studies, Reed et al. considers the outlook of renewable gas with most adoption driven by the transportation sector and conservative adoption in the power generation sector, whereas Colbertaldo et al. considers the using renewable gas as an energy storage vector in the power generation sector but fails to evaluate the impact from the transportation sector.

There also is another subset of works that focus on often overlooked challenges associated with the anticipated growth in renewables. Tarroja et al.^11^ suggest that climate induced variability requires greater dispatchable generation capacity, up by 6.3%. This variability also increases natural gas power plant downtime and consequently startup frequency, which has high emissions relative to operating at a steady level. This suggests more energy storage capacity needs to be deployed but Tian et al.^12^ compare operational carbon offset of battery energy storage system (BESS) accounting for upstream system procurement emissions and find that BESS at capacities absorbing 38–76% of daily generation has diminishing returns of carbon reduction in a scenario with 80% of electric supply being from solar and wind. Cases as early as 105% of daily average renewable generation would result in zero marginal environmental benefit, suggesting a BESS maximum deployment constrained by cost-effective carbon reductions. These types of findings help provide a guideline to narrowing down assumptions when modeling bulk system capacity in future scenarios motivated by environmental constraints.

Sinha and Brophy^13^ analyze the life cycle emissions of cross-sectoral decarbonization of the electricity and transportation sectors using renewable hydrogen production and vehicle technologies. By analysis of various case studies of electricity feedstock, they determine that a combination of policy, natural resources, and hydrogen technology advancements could overcome challenges with renewable intermittency to decarbonize both the electric and transportation sectors. However, Sinha and Brophy consider static use of the electricity feedstock and therefore do not consider the impact on the power generation sector from massive adoption of these fuel production pathways. Baik et al.^14^ analyze the effects of dispatchable low-carbon technologies and how it can cost-effectively replace significant portions of solar and batteries toward a carbon-free future. By considering various sensitivity cases, they identify the inclusion of more diverse technologies lowers the variability of future incremental system costs. The insight from this study suggests it would be beneficial to consider maintaining a small portion of traditionally natural gas assets in 2050 models assuming they are procuring low-carbon or net zero-carbon gas.

Renewable technologies can be incentivized in various ways to promote adoption. This interaction is further convoluted when one desires to consider the degree to which the local incentivizes can alter local adoption rates and how it may impact the global progression and adoption of a technology. These types of minor differences in assumptions used in models can propagate differing results. Mai et al.^15^, despite using the same inputs for three different commercial capacity expansion models, find that each model has differing results including: the capacity of renewable energy capacity installed, regional buildouts, curtailments, and least-cost optimized portfolios.

An emerging suite of studies co-optimizing carbon-intensive sectors, in other words, finding the lowest cost solution given certain environmental constraints, can be found in the literature^16–21^. Studies often set out to identify capacity expansion to meet carbon reduction goals or simulate a proposed portfolio meeting the same goals but with a focus on evaluating the dynamics. In our work, the latter focus is adopted to identify the value provided by electrolyzer systems that act as a sink for renewable generation. Rather than assuming modest adoption of renewable gas for the transportation sector and leaving a portion of the fleet unelectrified, this work poses the question of investigating the impact of massively adopting hydrogen as an energy vector to decarbonize the transportation sector beyond existing goals and investigating the resulting consequences for the power generation sector. Instead of assuming otherwise renewable curtailed electricity as the basis for electrolytic fuel production, this work aims to deploy additional renewable capacity for the explicit purpose of electrolytic hydrogen production. This hypothetical is investigated to garner insight on whether expanding the scope of existing renewable policy targets can be met with a lower cost solution by explicitly tackling a traditionally difficult to decarbonize portion of the transportation sector in conjunction with a deeply renewable electric grid. This is done by simulating an hourly year-long economic dispatch with consideration for varying levels of hydrogen demand, existing power transmission constraints, and regional differences in load and generation.

The objective is to minimize total power generation system cost whilst meeting the required electrolytic hydrogen fuel demand. The formula for this is a mixed-integer unit commitment optimization which is a function of fuel prices and generator characteristics while respecting key constraints such as balancing supply and demand, transmission line capacities, and generator limitations. There is no hard constraint to meet a renewable portfolio standard, rather the progression toward the state’s renewable goals is reflected by the modeled generator capacities as well as a carbon price. This is a more stable problem to solve as the necessity to account and optimize for a renewable balance with foresight of the entire year is too massive of a formulation. To accomplish this, a 2050 California resource portfolio is established by building from existing solutions in the literature aimed to decarbonize the power sector, but further augmented them to be able to meet the increased transportation sector hydrogen demand. Two levels of hydrogen demand from outside of power generation are used as an input for the hourly annual simulation, where additional renewable capacity is tuned to balance hydrogen production and consumption for all sectors. The adoption of offshore wind is the other key point of consideration, resulting in a total of four scenarios. By comparing the results of these scenarios, one can generate insights regarding the emission reductions and change in relative total costs from adopting offshore wind and hydrogen technologies. The approach is a compromise between capacity expansion models and least-cost dispatch solutions, so while the simulation of each scenario finds the least-cost dispatch given the presupposed capacities, the best solution must be evaluated by comparing the total system cost, the key metric and objective of the formalized least-cost dispatch problem. In addition, the total carbon emissions must be holistically considered from a sociopolitical standpoint.

The results of this work then provide a basis to understand the unitized cost per emission reduction basis by adopting these technology vectors and their economic efficiency relative to other approaches. The development of the resources which distinguish these four scenarios are largely due to uncertainty regarding the commercial maturation of these technologies and political support. This study is relevant because interest in viable pathways to deep decarbonization is increasing with support from agencies, corporations, and individuals around the world. Scientists, consultants, and the general public must be able consider the various approaches and the associated tradeoffs to achieve the shared goal of decarbonization across all sectors. California is a state which leads in renewable policy, research, and implementation. To this end, it is worthwhile to investigate a future hypothetical which expands the horizon of existing renewable policy, and the conclusions then generalizable to other states following suit.

## Methods

### Optimization model

Energy Exemplar’s PLEXOS production model built in the Microsoft .NET framework with SCIP as the solver is used to resolve the mixed-integer linear program unit commitment economic dispatch problem. The scope of this work is a multi-nodal annual simulation representing California’s power grid in 2050. The spatial resolution is established by furthering the discretization established in a California Independent System Operator (CAISO) 2020^22^ study modeling year 2026 and 2030 which evaluates the reliability of a low carbon emission resource plan portfolio. The existing list of generators, including scheduled retirements, up to 2030 act as a starting point for estimating 2050 capacities. One weather year was taken from one of the many generated renewable years used in their reliability study resulting in no forecast error**.** The optimization time horizon is set in daily intervals, meaning that the model has foresight within the same day to optimize unit commitments and passes on the state of the system as an input for the following day. The high electrification scenario in the 2021 SB Joint Agency Report is referred to estimate the necessary amount of capacity additions up to 2045. While the amount of natural gas capacity decreases, some capacity remains for reliability purposes. Note that this high electrification scenario being referenced assumes heavy electrification to optimally meet the considered emission goal and the remaining applications are left to other fuel sources. As such, all scenarios considered in this work proceed with the presupposition that anything that could have been renewably electrified is already adopted, with any additional hydrogen adoption occurring due to decarbonizing the remaining marginalized applications. Then, renewable capacity unique to this work is added to accommodate meeting renewable hydrogen demand via electrolysis. This will result in differing amounts of renewable generation capacity across considered scenarios. The electric load, excluding electrolysis, modeled is a 2035 hourly portfolio projection^23^ scaled to meet a 2050 annual projection total of 449 TWh^24^. This is a static electric load for end-use with an hourly resolution and does not include electricity used for charging batteries nor for electrolytic hydrogen production.

Two scenarios deploy 21 GW of offshore wind capacity. A National Renewable Energy Laboratory (NREL) report^25^ conducts a thorough cost-analysis for five study areas under commercial development consideration amounting to this 21 GW estimate. Although additional technical potential exists for California, this level of capacity is assumed to be sufficient for generating energy dynamics insights considering the diverse portfolio of technologies considered. While the buildout of such resources is still uncertain due maturing technologies, the possibility of its complementary generation profile to solar is of great value as it circumvents energy storage. Each of those two scenarios are then split by representing a low and high level of hydrogen demand each to evaluate the synergistic effects of minimizing the cost of the power generation sector with varying renewable hydrogen fuel production capacities. In short, a year-long hourly formal least-cost economic dispatch optimization dispatch problem is solved independently for four different scenarios under the presupposed generation capacity portfolios. The model is a least-cost economic dispatch problem, and any capacity changes are made exogenously to meet the electrolytic hydrogen demand constraint described later in this section.

### Energy system integration strategy

Due to the magnitude of the formulation, one tradeoff taken is inputting system characteristics that are fixed (e.g., transmission line loading limits, gas generator heat rates) to focus on fuel commitment dynamics throughout the year as opposed to identifying marginal system improvements. Electrical nodes are connected by power lines to reflect existing transmission capacity. Gas nodes are co-located with electric nodes, connected by hydrogen pipelines modeled without capacity constraints to allow the flow of hydrogen to a central gas storage inventory. This is done under the assumption that gas transmission is sufficient in throughput capacity and the physical buffering of gas allows greater leniency in times of hydrogen gas demand and supply mismatch. It is likely that any gas transmission constraints would be less constraining compared to the electric transmission system constraints due to inherent storage available in the linepack, making gas transmission the most cost-effective bulk energy transmission approach. The cost of storing gas relative to the levelized cost of electricity is order of magnitudes lower, especially for renewable high penetration renewable system. These factors regarding additional renewable energy transmission capacity costs are explored and discussed further in Thai and Brouwer^26^ to justify modeling zero gas storage cost. The fluid dynamics of pipeline transport and development feasibility are outside the scope of this work but are recommended for future high renewable penetration analyses.

The economic dispatch problem aims to minimize total system cost. Renewable generators (i.e., solar, wind, geothermal, some hydropower) must commit or otherwise be curtailed if load is sufficiently met and storage systems are fully loaded. Several gas turbine generators are also designated as must commit for reliability reasons. These are converted to hydrogen gas turbines to facilitate the carbon neutrality transition despite Colbertaldo et al.^10^ only considering fuel cells only for re-electrification in a 100% renewable case. However, majority of additional hydrogen-fueled generators are fuel cell generators due to their lower emissions, higher electrical efficiency, and quick start-up characteristics, driving a variable hydrogen demand. Ultimately, hydrogen gas turbines are designated as must run units that are able to provide system reliability due to their operational experience. To contrast, fuel cells are deployed with the advantage that they will be sparsely dispatched to supplement generation as their degradation is proportional to operation time. In reality, the capacity balance between the two different fuel cell generator technologies is subject to change dependent upon actual technology adoption experience and social support. Converting gas turbines from natural gas to hydrogen may pose a lower financial risk utilizing an otherwise stranded asset or it may be possible that fuel cell plants completely replace hydrogen gas turbine plants due to higher electrical efficiency considering fuel costs. The authors opt for a balanced approach in favor of focusing on the effects of considering further hydrogen adoption outside of power generation. The three major storage assets are (1) BESS, (2) electrolyzers and (3) pumped hydroelectric storage facilities.

Establishing a penalty price on curtailment incentivizes excess electricity going toward storage rather than being curtailed. This promotes both daily energy shifting and seasonal energy storage. In reality, market participants submit economic bids and actively make decisions based on price signals (e.g., real-time price of electricity is zero or negative), however in a least-cost economic dispatch optimization the dispatch is done so assuming the bids are all submitted at marginal cost for solving efficiency. This optimization seeks an objective lowest system cost approach as opposed to an approach which represents market participants attempting to capitalize on realtime imbalances. A penalty price on unserved energy, modeled at orders of magnitude higher cost than any marginal generator, is used to balance supply and demand for all hours to ensure solution convergence. The model dispatches batteries to charge and discharge with foresight of the time horizon (i.e., one day at a time). However, a major challenge is when the batteries lack the foresight to charge and retain state-of-charge across multiple days. This is necessary as days without much solar production would exacerbate scarce supply during the traditional daily evening ramp and peak load. A solution to this pitfall would be to increase the time horizon optimization to be able to look further than one day at a time. However, this would significantly increase the simulation time considering the scope of this analysis. In addition, forecasting is frequently subject to error.

In practice, it is in the best interest for batteries to charge and discharge daily to meet resource adequacy requirements where price signals are secondary in the overall operational strategy. As such, the penalty price on curtailment is necessary to reflect unquantified but essential system reliability. Further, penalty prices are used to prioritize charging batteries for short-term energy storage relative to committing to electrolytic hydrogen production. This is done due to the higher roundtrip efficiency of batteries to cost-effectively shift energy in the short term in addition to the flexibility to store energy seasonally as hydrogen. Without these penalty prices, the solution will not converge due to insufficient generator availability with batteries lacking state-of-charge and insufficient electrolytic hydrogen production. This is because the marginal cost of curtailment to a renewable power plant is zero. The basis of this evaluation considers the presupposed question of investigating whether aggressive hydrogen adoption relative to a conservative adoption in addition to deep electrification would result in lower total system costs due to cross-sector decarbonization synergies. As such, the penalty costs that result in charging batteries and producing electrolytic hydrogen are necessary to reflect the trend of development and operation per state policy.

While there are major challenges in pinpointing an optimal distribution of renewable generation and storage technologies with a far outlook, this study provides a representative snapshot of an annual dispatch if one were to accept the (1) 2045 generator capacity established in the high electrification scenario of the California Energy Commission (CEC)^24^ as the lowest-cost portfolio for meeting the power generation sector load and (2) the deployments (i.e., solar, offshore wind, fuel cells, and electrolyzers) to accommodate supplemental massive amounts of fixed cross-sectoral hydrogen demand as well as its variable usage in power generation are operating in an economically-viable manner.

Total system load reported in the results section of this paper will include electrical loading of storage systems in addition to the 449 TWh of retail electric load. The fixed non-power generation sector annual hydrogen demand is 167 TBtu and 563 TBtu for the L-scenarios and H-scenarios, respectively. While generally, watt-hours are used to represent energy units of electricity and British thermal units are used to represent energy units of gas, this hydrogen demand expressed in watt-hours is 49 TWh and 165 TWh for reference, respectively. The low and high hydrogen demand outside of power generation usage is static, split evenly throughout the year, and corresponds to the low and high 2050 annual projection in Reed et al.^9^, with usage comprising mostly for the transportation sector at nearly 70%, but also including heating applications at roughly 25%, and other industrial uses. In other words, the non-power generation hydrogen load is assumed to be the same for every hour of the year. This assumption could be improved upon, especially as heating loads are expected to peak in colder months coinciding with minimal electrolytic hydrogen production and consequently exacerbating the required storage capacity but is left to future work. The hydrogen demand for power generation is determined as part of the solution on a per scenario basis. These numbers are summarized in the Table 3 in the Results. The only constraint regarding hydrogen gas storage is that the start of the year amount must be equal to the end of the year amount. This is accomplished by modeling an unconstrained maximum and minimum value. If this continuity constraint is not met, the entire annual simulation is reiterated with additional renewable generation and electrolyzer capacity is increased or decreased monotonically until the difference between starting and ending volumes is within a quarter-percentage point of working volume. The daily optimization assumes there is no shortage of fuel supply when dispatching hydrogen-fueled generators. An arbitrary starting volume is established as the total amount of storage is unknown, but the intra-annual working volume can be interpreted to be in addition to the cushion volume needed in real gas operations and is calculated as the difference between the annual maximum and minimum volumes.

To summarize, the electric demand is modeled to represent the entirety of the state, and consequently any hydrogen usage used for power generation is as well. However, hydrogen demand outside of power generation (e.g., light-duty transportation, freight, and industrial processes) is assumed to not have major seasonal differences relative to the seasonal consumption of hydrogen in power generation. This is intentional as the focus of this work addresses the challenges and impact on seasonal renewable electric power supply dynamics enabled by an increased level of hydrogen adoption but is worthwhile to investigate in future work. It should be noted that the basis of this work is expanding renewable capacity beyond serving the non-electrolytic consumer load and therefore any additional capacity is deployed with the purpose of fuel production to meet a fuel demand constraint. To model marginally deployed generators and electrolyzers as flexible load could be enabled in this scenario by considering the opportunity cost to export to other balancing authorities and is recommended for future work. It should be emphasized that the modeling of these assets in a high renewable penetration scenario result in different benefits than from deploying electrolyzer systems in the short-term, e.g., by providing flexible load services as discussed in Ruggles et al.^27^.

### Spatial discretization

**Figure 1:**
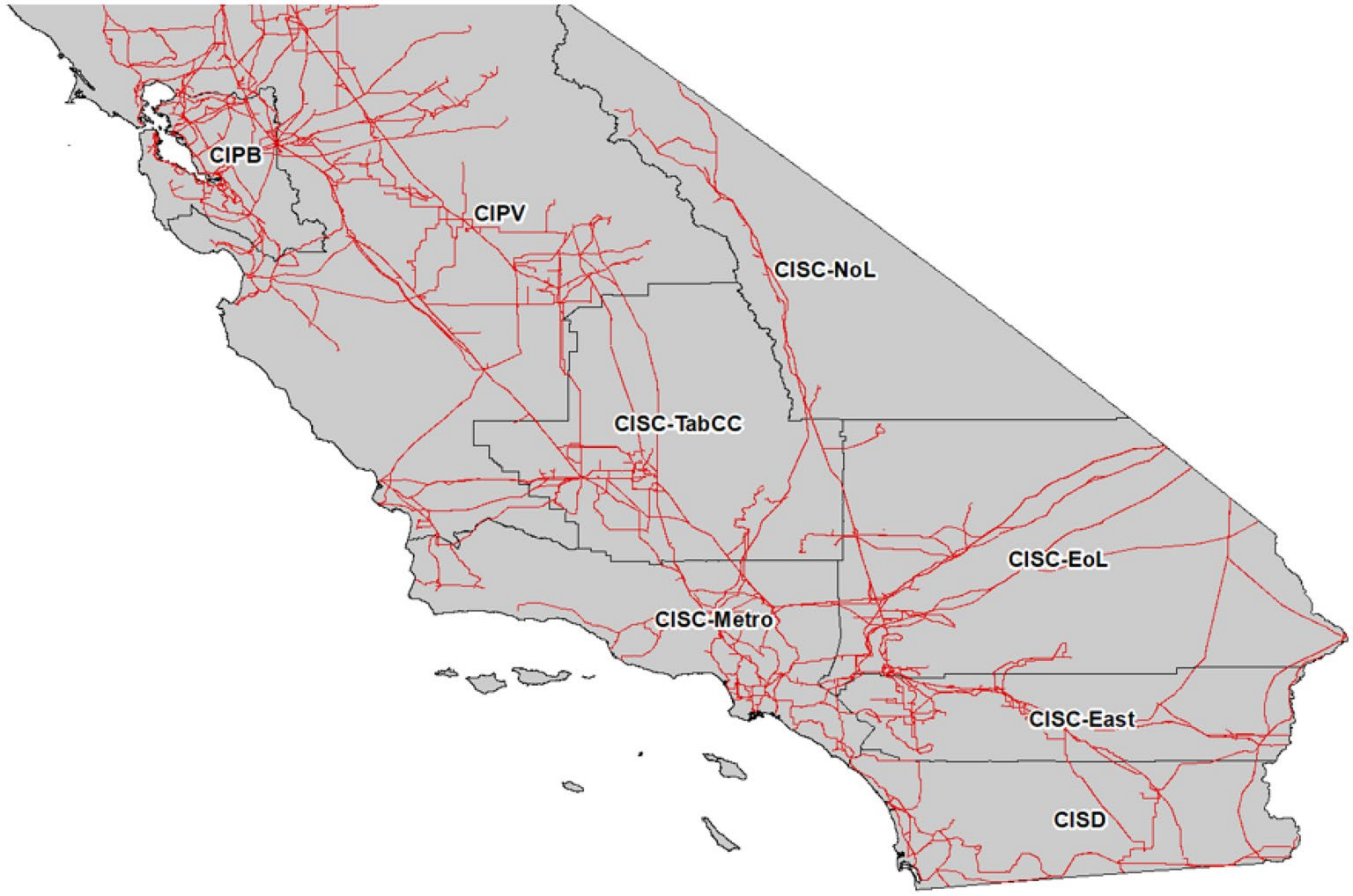
Spatial discretization for the California electric grid model. Nodes are formed by grouping counties overlaid with 115 kV and higher existing transmission electric lines. Image is created with ESRI’s ARCGIS Desktop version 10.8.2. (https://www.esri.com/en-us/arcgis/about-arcgis/overview).

Spatial nodes and transmission system are laid out CAISO 2020^22^ as in but the Southern California Edison (SCE) service territory is further broken down into five nodes guided by CAISO 2019^28^ nominally: (1) CISC-Metro representing the metropolitan area of SCE, (2) CISC-East representing the eastern area of SCE, (3) CISC-EoL representing the area east of Lugo, (4) CISC-NoL representing the area north of Lugo and, (5) CISC-TabCC representing the Tehachapi and Big Creek area as depicted in Fig. 1 below. This results in a total of 9 nodes, including one to represent outside of the state. Figure 1 does not capture the entirety of the CAISO jurisdiction, with CIPV being truncated, but rather is framed to highlight the areas represented by each of the nodes. Exports to OOS are possible and are modeled at the marginal production price plus 20 $/MWh for transmission, however, generally there is already a shortage of excess electricity to meet the electrolytic hydrogen fuel demand.

Transmission line capacities also follow the CAISO study and transmission line capacities resulting from the CISC region breakup are established by evaluating existing corridor circuits^29^. The loading limits used in this work are summarized in Supplementary Table 3. CISC and CISD are used nominally in this work to roughly represent the SCE and San Diego Gas and Electric (SDG&E) investor-owned utility (IOU) service territories, respectively, and both CIPV and CIPB comprise the Pacific Gas and Electric (PG&E) service territory. The nodes are established on a county basis resulting in some simplification in spatial nodes’ bordering areas and should not be interpreted as necessarily associated with an IOU.

Imports and exports are defined as electricity exchange between regions in the model. By this definition the total amount of imported electricity is equal to the total amount of exported electricity as the external node, representing OOS, is included. The transmission is modeled as a lossless direct-current optimal power flow problem.

### Generator costs

The total cost of generation consists of three factors that establish the merit order of dispatch: fuel costs, emissions costs, and supplementary generation costs. Three of the most prominent fuels with explicit costs in this work are natural gas, biogas, and hydrogen gas. Natural gas fuel prices in the future are quite uncertain, however, higher natural gas prices would imply greater dependence on renewable power generation and fuels. As such, the decision to use frozen natural gas prices as used in the CAISO 2020^22^ simulation would implicate a more conservative adoption of renewable resources to meet the scenario demands. The utilized natural gas prices have regional and monthly differences but generally range from a minimum price of 3.3 $/MMBtu to a maximum price of 5.5 $/MMBtu. Note that this range falls into the median of EIA Henry Hub price estimations in 2050^30^, so that the expected cost would be higher to deliver the produced natural gas to California. Biogas is modeled by feedstock with prices ranging from 3 to 7 $/MMBtu based upon a Duke study evaluating the supply cost curve in the United States^31^. The cost of hydrogen is 33 $/MMBtu established by conducting a cash flow analysis as done by Lazard^32^ but with a 25% electrolyzer capacity factor (CF). This CF, which can significantly affect hydrogen price, is established through iterative simulations that indicate this value is typical of an electrolyzer operating solely for green hydrogen production with dominantly solar electricity as feedstock. To this end, the electrolyzer capacity is iterated to achieve this capacity factor simultaneously as additional renewable capacity is iterated to balance annual renewable hydrogen demand. The approach of the simulation requires additional deployment of renewable and electrolyzer capacity if the electrolytic hydrogen demand is not met. This then in turn affects how the electrolyzer would operate. In other words, the hydrogen price modeled here is a converging endogenous result of the optimization.

The cost of carbon modeled is 250 $/MTCO_2_e^33^. Carbon emission rates are tied to the fuel, with natural gas being 117 kg of CO_2_ per MMBtu of fuel consumption^34^. Similarly, for biogas this number is 50 kg/MMBtu^34^ and zero for hydrogen, as all hydrogen production is electrolytic in the current analyses. Note that the reported results are tailpipe or exhaust carbon amounts, but the upstream production of these fuels are not represented. Depending upon the production pathway of these fuels, the life-cycle emissions could be net-zero or negative, qualifying them to be “renewable” fuels. While some portion of natural gas can be procured in this manner, the focus of this work proceeds with the motivation to investigate electrolytic hydrogen as a dominant energy vector for decarbonization in response to renewable natural gas supply concerns^31^.

Supplementary generation costs effectively encapsulate all other costs including annualized costs from the capital expenditures and typical VO&M costs. For the most part, these costs can be broken down by fuel technology type. Natural gas, biomass, and nuclear power plants are assumed to be fully depreciated and are taken from an assumed low capacity factor operation^35^ minus fuel costs. The lower range of wind from the same source, 26 $/MWh, is taken to represent the development of high renewable potential sites as well as out-of-state excess wind imports, which is also quite similar to existing imports of renewables from the Pacific Northwest. A new gas turbine combined-cycle plant capital cost is used to represent a new hydrogen gas turbine combined-cycle plant capital cost at 24 $/MWh. Hydropower and geothermal costs come from IRENA’s historical evaluated costs^36^. Behind the meter PV (BTMPV) costs are taken from NREL’s annual technology baseline^37^. BESS costs are taken by removing the cost associated with PV from the PV paired with 4-h batteries in Lazard’s evaluation of the cost of storing energy^38^. Batteries are modeled to have an 85% roundtrip efficiency. In summary, these supplementary generation cost numbers are estimates based on an assumed capacity factor in addition to annualized hardware costs to arrive at a per MWh cost and is only one of the three parts that will affect the cost of the plant supply energy. These estimates are summarized in Table 1.

**Table 1:** Supplementary generation cost.

Generator Type	Supplementary Generation Cost ($/MWh)	Source
BESS	85	^38^
Biomass	10	^40^
BTMPV	40	^37^
Geothermal	50	^36^
Fuel Cell	42	^41^
Hydrogen GT	45	^40^
Hydropower	10	^36^
NG GT	10	^40^
Nuclear	29	^40^
Offshore wind	91	^25^
Utility-scale solar	35	^40^
Onshore wind	26	^40^

Supplementary generation cost in addition to fuel costs and emissions costs make up total generation cost for each technology type.

Fuel cell generators are modeled with a supplementary generation cost necessary to offset capital costs from a system with a 10% CF operation, 42 $/MWh. This is calculated conducting a cash flow analysis achieving a 12% after-tax rate of return per^20^ assuming a stack life of 40,000 h, 1200 $/kW initial installation cost and a 425 $/kW stack replacement cost. This supplementary generation cost, in addition to the cost of fuel, represents the cost of generation for the fuel cell generators. The dispatch within the scenarios is then reviewed and the capacity heuristically increased until the CF decreases to roughly 10%. A CF higher than 10% suggests that this level of revenue would be sufficient to justify economically deploying fuel cell system capacity at this cost and operation. This is to balance the necessary power generation needed to meet nighttime loads without overbuilding fuel cell capacity with low CF that may be deemed economically unviable. In short, too little fuel cell capacity would result in an unsolvable problem and too much fuel cell capacity is assumed to be uneconomical for investors due to exponentially increasing levelized hardware costs due to infrequent operation. Despite this, the fuel cost associated with generating electricity with fuel cells comprises a majority of the cost and slight differences in these supplementary generation costs are inconsequential to the dispatch behavior between scenarios. Future work is recommended to investigate sensitivities to this CF which directly impacts (1) the fuel cell capacity deployed, (2) consequently the additional primary renewable electricity generation and electrolyzer system capacity required to balance the hydrogen gas consumption, and (3) the resulting reduction in carbon emissions due to reduced dependence upon natural gas fueled power plants.

Utility-scale solar and offshore wind capacity primarily deployed in addition to the 2045 CEC^26^ portfolio is treated slightly differently. Due to the multi-GW scale capacities considered, the cost of interconnection also needs to be considered to fairly compare the two. The generation portion for utility-scale solar is 30 $/MWh^36^ and 63 $/MWh for offshore wind^25^. However, offshore wind would likely need additional cost of interconnection which is thought to range from 14 to 41 $/MWh^36^ or an average of 28 $/MWh, which is used in this study. The equivalent for low CF spur-lines delivering new build solar is modeled at 5 $/MWh^39^. The sources for these costs are summarized in Table 1 below.

### Generator capacities

The CEC Report^24^ anticipates 55 GW of BESS capacity additions in the 2045 core and high flexibility scenarios which results in a total of roughly 57 GW. The total BESS capacity modeled in this work is also 57 GW with the difference from the capacity modeled in CAISO 2020^22^ distributed along with solar, electrolyzer, and fuel cell capacity, geospatially sited based upon existing capacity-weighted CEC solar power plants above 10 MW found to be: East 24%, EoL 20%, Metro 16%, NoL 0%, and TabCC 40%. This is done as deploying BESS co-located with solar resources is mandated by the state. In addition, electrolyzers are thought to help sink excess renewable generation and fuel cell generators can meet nighttime loads where existing transmission is available as they can be co-located with BESS and solar sites. While this describes the location the additional renewable capacity is sited, the total capacity differences in the four scenarios are primarily due to meeting the varying hydrogen demand both in and out of the power generation sector. The distribution of capacities is both a preliminary task to the annual simulation and iterated upon as previously described. Namely, the electrolyzer, solar, and fuel cell capacities are incrementally increased in the iterative process, whereas all other capacities are generally static inputs to the optimization. These total capacities are summarized below in Table 2.

**Table 2:** Installed system capacity.

	L-S	L-W	H–S	H-W	
	Installed Capacity (MW)				Note
Electrolyzer	80,000	55,500	146,500	114,700	Iterative adjustment per “Methods” section
Fuel cell	26,000	6,000	23,500	6,000	Iterative adjustment per “Methods” section
Solar	150,620	95,500	212,610	163,490	Iterative adjustment to balance hydrogen demand in addition to adjusting 2030 model^22^ with CEC report projections^24^
Wind	14,770	35,940	14,770	35,940	Consideration for offshore wind in addition to adjusting onshore 2030 model^22^ with CEC report projections^24^ in addition to offshore wind
BESS	57,440	57,440	57,440	57,440	Adjusted 2030 model^22^ with CEC report projections^24^
Biomass	50	50	50	50	Adjusted 2030 model^22^ with CEC report projections^24^
BTM solar	34,250	34,250	34,250	34,250	Adjusted 2030 model^22^ with CEC report projections^24^
Geothermal	1850	1850	1850	1850	Adjusted 2030 model^22^ with CEC report projections^24^
Hydrogen GT	8430	8430	8430	8430	Converted reliability designated natural gas plants from 2030 model^22^
Hydropower	11,070	11,070	11,070	11,070	Adjusted 2030 model^22^ with CEC report projections^24^
Natural gas GT	28,820	28,820	28,820	28,820	Adjusted 2030 model^22^ with CEC report projections^24^
Nuclear	4210	4210	4210	4210	Adjusted 2030 model^22^ with CEC report projections^24^

Utility-scale solar, offshore wind, and electrolyzer system capacity for hydrogen production vary by scenario.

### Carbon and pollutant accounting

The difference between the cost of fuel and the amount of carbon emissions associated with the fuel displaced by renewable hydrogen amongst the H-scenarios and L-scenarios is estimated, allowing the four scenarios’ total cost and emissions to be compared on the same basis. Hydrogen demand in the L-scenarios primarily meets fuel cell electric light-duty vehicle (LDV) demand, thus displacing primarily gasoline per Reed et al.^9^. However, the hydrogen demand in the H-scenarios displaces a mixture of gasoline, diesel, and natural gas used for fuel cell LDVs, medium-duty vehicles, heavy-duty vehicles, and industrial applications. Additionally, Bureau of Transportation Statistics 2030 emission factors^42^ are used to estimate pollutant emissions factors. The offset market for NOx, CO, and particulate matter in California is not as mature as the offset market for CO_2_ resulting in low trade volume and high price volatility. Despite this, 2500 $/tonne, 25,000 $/tonne, and 15,000 $/tonne are used for CO, NOx, and PM2.5, respectively, as representative values based upon market data averages from 2017 to 2018^43^. These values are expected to be conservative as the price in 2050 could increase as allowances change in the future akin to the CO_2_ market.

### Other considerations

Wind and solar generation profiles per node are modeled using a fraction of CAISO aggregate totals. Consequently, the generation profile is the same regardless of the spatial node, however, existing assets in the state portfolio are evaluated for their physical location and assigned to the corresponding modeled node. Additional renewable capacity is deployed by propagating the existing power plant ratio. For example, if 60% of existing solar power plants are in an eastern county in California, then any additional capacity will be deployed similarly. This acts as a proxy for resource and transmission availability as commercial development interest has uncertainties not evaluated in this study. Reserve demands are used as done in CAISO 2020^22^ with non-spinning and spinning requirements being 3% of load per region. Thermal power plants are modeled with previously CAISO established outage rates, maintenance rates, outage times, time to repair, startup costs, and associated fuel offtake amounts at start. Fixed heat rates are established across the fleet by categorizing generators as peaker plants, baseline generators, or an intermediary to reduce solution convergence time.

Some out-of-state (OOS) resources are designated must-take dedicated imports, which share a maximum import capacity with any additional purchases. This reflects the joint ownership of assets utilities have that are outside of the state boundaries. The remainder of imports from OOS reflect the exchange of excess renewables between states that are representative of current operations and expected to be the status quo as other states deploy more renewable assets. The maximum import limit at any given hour is 7.8, 13.5, and 4.2 GW for the PG&E, SCE, and SDG&E areas, respectively, representing the physical transmission capacity limits at interties. Exports are possible and are modeled at the marginal production price plus 20 $/MWh for transmission, however, generally there is already a shortage of excess electricity to meet the electrolytic hydrogen fuel demand.

## Results

### Scenarios overview

Four scenarios are nominally referred to in this study as the L–S, L–W, H–S, and H–W scenarios with the first letter denoting the level of fixed hydrogen demand input (L = low, H = high) and the latter denoting the installation of offshore wind capacity (S = no offshore wind, W = 21 GW offshore wind). Though these scenarios are not meant to represent absolute possible edge cases, the comparison of these scenarios are meant to provide insights derived from the general trend of having more offshore wind or having increased electrolytic hydrogen production, which inherently requires greater levels of renewable generation capacity whether it be solar or offshore wind.

**Figure 2:**
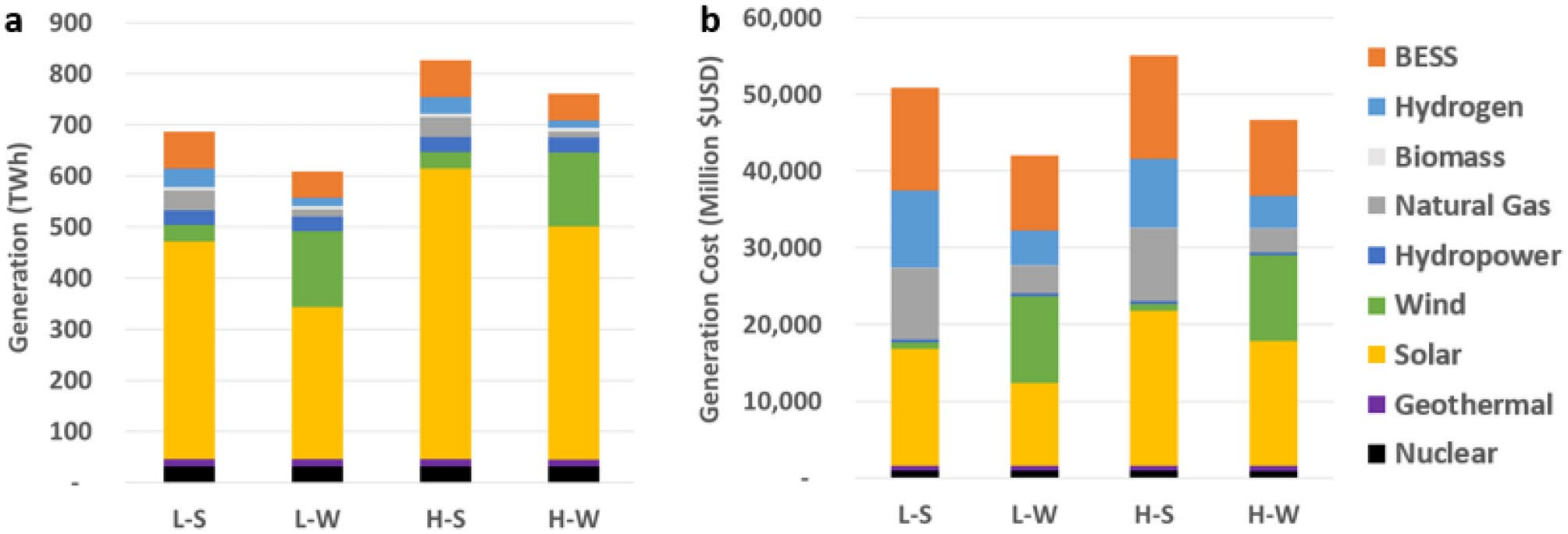
Total scenario generation and cost. (**a**) Annual generation and (**b**) generation cost by scenario and fuel type.

Comparing percentage of each technology meeting load versus the cost will help build intuition of why certain scenario total system costs are different. Solar makes up 60%, 46%, 67%, and 58% of total generation for the L–S, L–W, H–S, and H–W scenarios, respectively. The relatively low cost of solar generation is evident from the generation dispatch and cost results are presented in Fig. 2 [comparing total solar (a) generation and (b) cost]. On the other hand, the cost of energy storage is evident as well as BESS which makes up 11%, 9%, 9%, 7% of total generation but account for 26%, 23%, 24%, and 21% of the total cost for the L–S, L–W, H–S, and H–W scenarios, respectively. Similarly, hydrogen-fueled generators make up 5%, 3%, 4%, and 2% of total electric generation, but account for 20%, 11%, 16%, and 9% of total system cost for the L–S, L–W, H–S, and H–W scenarios, respectively. These four technology types are the ones that differ the most between the scenarios. Meeting the same electric and hydrogen demand, the wind scenarios are more cost-efficient with both equivalent wind scenarios costing 17% and 15% less in the low and high hydrogen demand scenarios, respectively. This can be largely attributed to the reduced need for BESS to shift solar production and instead relying on offshore wind to meet load directly. Note that the generation totals in Fig. 2 are for meeting electric load directly, including electrolyzer load. This means that any renewable electricity that charges batteries is not counted twice.

### Carbon emissions

**Figure 3:**
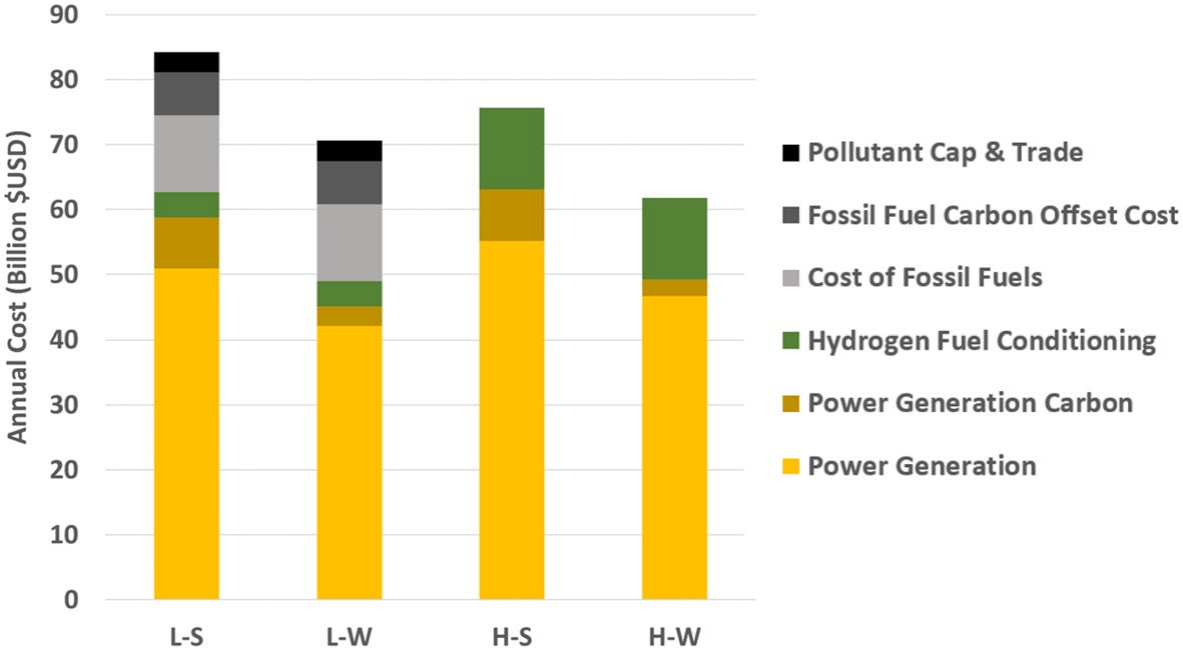
Comparison of total scenario costs. Electricity generation costs, hydrogen transport and fuel conditioning costs, fossil fuels costs and associated pollutant cap and trade value are considered.

Aside from cost, total carbon emissions is a key metric to evaluate. The L–S and H–S scenarios result in an annual CO_2_ emission total of 31 MMT and 32 MMT, respectively. The L-W and H-W scenarios result in 12 MMT and 10 MMT CO_2_ emissions, respectively. The high hydrogen demand scenarios have less carbon emissions than their low hydrogen demand counterparts because there is greater solar capacity deployed—reducing the need for flexible generators in the off-peak solar generation hours. In addition, the high hydrogen demand also displaces natural gas, diesel, and gasoline usage in the other sectors outside power generation, primarily heavy-duty transportation. Figure 3 provides a direct comparison between scenarios by accounting for the cost of purchasing emissions offsets for any fossil fuel use that remains in the scenario and the incumbent fuel costs themselves, which are avoided with increased usage of hydrogen as a renewable fuel in both the power and transportation sectors. This then allows a normalized cost comparison between the scenarios by bridging the difference in total carbon emissions between scenarios. Note that deep electrification is used in all scenarios of this work. Note also, the fossil fuels being displaced by hydrogen are primarily difficult to electrify applications such as: high temperature process heat; ammonia, chemicals, steel, and cement production; long-haul freight transport and shipping. Therefore, both the low and high hydrogen demand scenarios presented in this work only use hydrogen in applications that require hydrogen for decarbonization.

The carbon reduction between the low and high demand scenarios achieved by using electrolytic hydrogen outside the power generation sector is 26.6 MMTCO_2_e. This number is calculated by considering the difference in the amount of gasoline, diesel, and natural gas that the electrolytic hydrogen would displace between the high and low hydrogen demand scenarios in the other sectors, considering previous emission factor assumptions^34,42^. The low–high total carbon emission difference as described in the previous paragraph, (i.e., 31 vs. 12 MMT and 32 vs. 10 MMT) differs slightly from this 26.6 MMTCO_2_e figure due to total carbon from the power generation sector. The cost of carbon is key for comparing the low–high pairs as the cost of carbon and pollutants from displaced fuel use outside of power generation makes up 12 and 14% of the total cost for the L–S and L–W scenarios, respectively.

If the price of carbon was 38 $/MTCO_2_e instead of the actual modeled and expressed 250 $/MTCO_2_e in Fig. 3, the total system cost of L–W and H–W would be the same. In short, the total cost between respective wind-dominant and solar-dominant pairs are quite similar even without accounting for the environmental value premiums. This suggests that the synergy between producing more electrolytic hydrogen (i.e., in the high hydrogen demand scenarios) and the additional renewable capacity deployed can also reduce carbon emissions largely in the transportation sector cost-effectively, without depending on an increasing cost of carbon, when framing the solution to holistically include multiple sectors.

While the low–high pair for wind has a 14% carbon reduction from power generation, the H–S scenario has 2% higher carbon emissions from power generation than its L-demand counterpart. This suggests that despite meeting a higher electrolytic hydrogen demand outside of the power generation sector, there is only a slight benefit to the power generation sector in the form of 2% of carbon emission reduction at an increased cost. This is only explained by the slight differences in fuel cell capacity deployed. The L–S scenario has 26 GW of fuel cell systems operating at an 11.3% capacity factor (CF) whereas the H–S scenario has 23.5 GW operating at a 10.6% CF. More electricity from fuel cell generators results in a lower dependence on natural gas fueled generators. The two wind scenarios both have 6 GW of fuel cell capacity installed with a 10.0% CF in L–W and an 8.0% CF in H–W. This capacity is sufficient to complement the addition of offshore wind meeting nighttime loads and having more opportunities to run on days with less solar relative to the scenarios without offshore wind. As seen previously, the cost of hydrogen fuel cells is higher relative to primary renewable generation despite its ability to be more flexible than BESS, further strengthening the argument for offshore wind development to reduce the need for both types of energy storage.

Fuel cells operate with more capacity at a higher CF in the L-demand scenarios than the H-demand scenarios because the higher capacity of solar installed in the H-demand scenarios provides more electricity to load in characteristically low solar production hours (i.e., mornings, evenings, and winter months). In other words, deploying more fuel cell capacity creates a virtuous feedback loop where the opportunity to dispatch between deployed fuel cell systems shrinks, but also the additional solar capacity deployed to balance hydrogen production also slightly reduces the need for fuel cells to dispatch in winter months**.** In short, major carbon emission reductions outside of the power generation sector can be achieved with electrolytic hydrogen while also synergistically reducing the need for flexible generators, most notably during times of low solar production. While this phenomenon may slow the actual deployment of fuel cell capacity resulting in a slight increase in carbon emissions in the power generation sector, the reduction from hydrogen use in the transportation sector and the net overall carbon emissions reductions are an order of magnitude higher.

### Generator dispatch

Dispatching natural gas generators with the carbon premium still occurs primarily due to the lower fuel price. This could change if the price of hydrogen, which makes up majority of the hydrogen gas turbine levelized cost, shrinks, or the cost of natural gas fuel is higher than the maximum modeled 5.5 $/MMBtu. For reference, at the end of 2022 the cost of natural gas for California doubled this modeled price for months due to reduced oil supply with increased global conflict. The cost of hydrogen is dependent upon the electrolyzer CF and the cost of feedstock electricity. A reduction in capex and electrolyzer stack replacement of 50% is found to reduce the levelized cost of hydrogen (LCOH) from 4.42 to 3.69 $/kg whereas doubling the CF with offshore wind would increase the price from 4.42 to 6.04 $/kg. While more offshore wind capacity can be installed to increase the CF of the electrolyzer, the average cost of feedstock electricity would increase. In this case, the complementary dynamics of offshore wind and solar would not necessarily decrease the price and promote more hydrogen fueled power generation, though if another source of cheap renewable electricity is available when solar is not this would further decrease the hydrogen cost. If the CF could double at the same solar price perhaps by similarly priced renewable imports from OOS, the LCOH reduction would be the same as the 50% reduced capex case. By far, the most sensitive single factor to the LCOH is the price of feedstock electricity, where if the LCOE is reduced to 15 $/MWh from 35 $/MWh, the resulting LCOH would be 2.75 $/kg.

Due to the nighttime generation of offshore wind, less hydrogen is used for power generation in the evenings throughout the year with BESS being nearly sufficient in the wind scenarios. The annual total power generation from BESS is 72 and 73 TWh for the L–S and H–S scenarios, whereas it is lower in the L–W and H–W scenarios at 52 and 53 TWh, respectively. The annual hydrogen-fueled power generation in the L–S and H–S scenarios is 36 and 33 TWh, respectively, whereas for the L–W and H–W scenarios this number is 15 and 14 TWh, respectively. Seasonal differences can be seen as BESS operates providing roughly the same amount of power every month in the solar scenarios and provides slightly more in the winter and autumn in the wind scenarios. Power generation using hydrogen is skewed toward the autumn and winter months for all four scenarios but more evidently so in the solar scenarios (see Supplementary Fig. 1).

**Figure 4:**
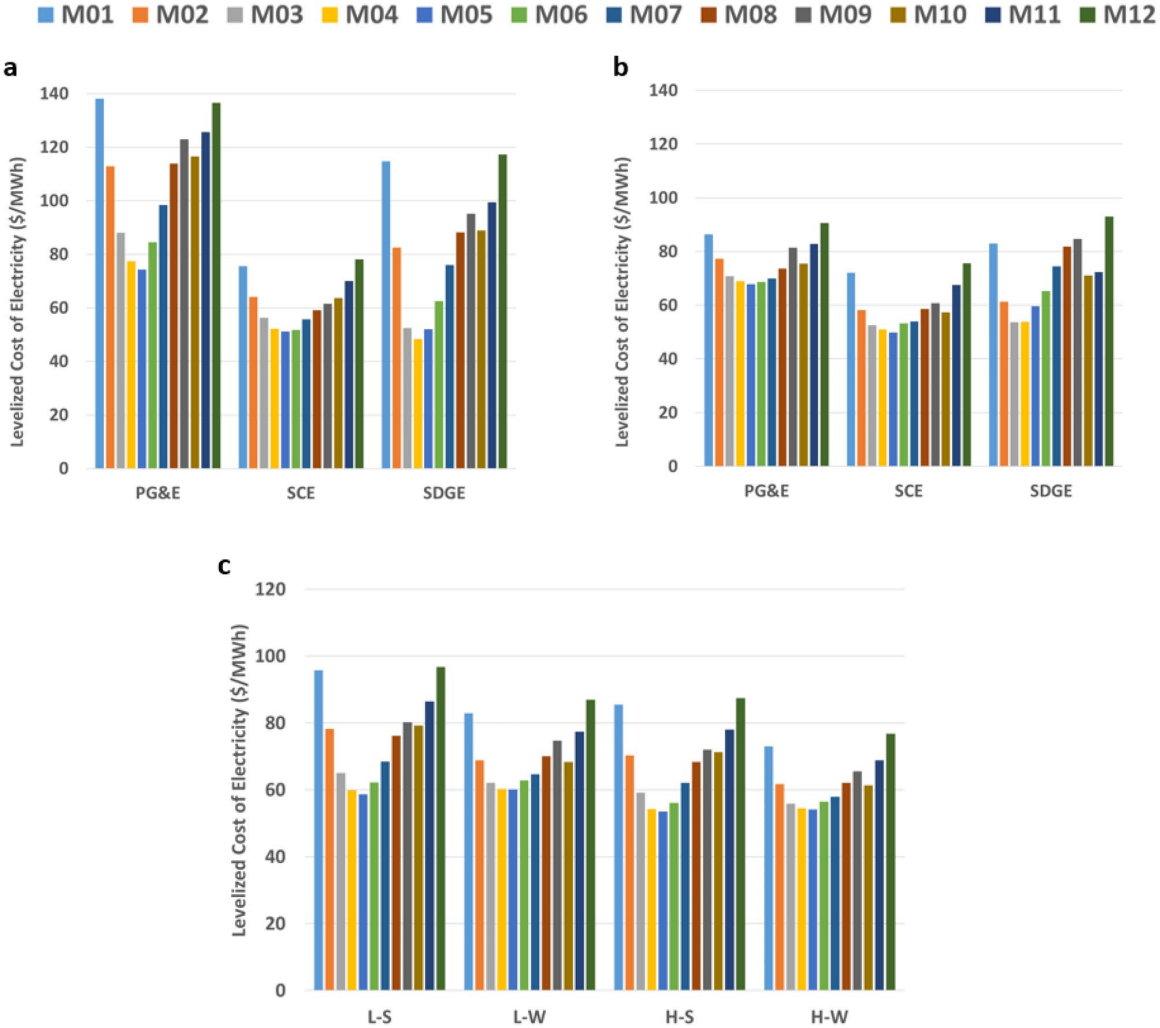
Levelized cost of electricity. (**a**) Solar scenarios by region and month, (**b**) wind scenarios by region and month and (**c**) all scenarios by scenario and month.

The LCOE between solar cases and between wind cases does not significantly change, thus Fig. 4 presents a subplot for each pair; however, with the deployment of offshore wind the levelized cost is much more even from month to month. Subplot a) and b) aim to illustrate the impact of having offshore wind affect each IOU differently, as offshore wind is primarily located in PG&E service territory and much of the solar capacity is located in the other two IOU service territories. Subplot c) considers the entire state and provides a snapshot of the different monthly costs for the total system, primarily driven by seasonal renewable dynamics. Without offshore wind capacity, the solar scenarios are more reliant on flexible generators. Similarly, the magnitude of solar generation is significant and is reflected by the relatively lower costs in the SCE region, as this is where most solar and battery capacity is located.

In the solar scenarios, natural gas power plants meet 5% of the total annual load, whereas this figure drops to 2% for both wind scenarios. Hydrogen used in both gas turbines and fuel cell systems meets 5% and 4% of the total annual load for the L–S and H–S scenarios, respectively, whereas this figure is 3% and 2% for the L–W and H–W scenarios, respectively. BESS meet 11% and 9% of the total annual load in the L–S and H–S scenarios, respectively, as opposed to 9% and 7% of load in L–W and H–W scenarios, respectively. In part, because of the higher utilization of BESS and hydrogen-fueled generators in solar scenarios, the overall levelized cost across the board is higher than in the corresponding months in the wind scenarios. These percentages of load are lower than other studies as other studies do not include the cross-sectoral transportation demand leading to large loads associated with electrolytic hydrogen production. The annual capacity factor of generators by fuel type can be found in Supplementary Table 1. The electricity used for electrolysis compared to total load is 28%, 21%, 41%, and 39% for L–S, L–W, H–S, and H–W, respectively. This is a massive amount of electricity which also provides opportunity to use electrolyzers as a dispatchable load to help provide realtime grid reliability. This benefit, along with consideration of variable weather and power plant outages at gigawatt-scale electrolyzer capacity is strongly recommended for future work.

Unless offshore wind costs are more expensive than expected, the ability to directly meet load without storage is ultimately more cost-effective than shifting energy daily in a solar-dominant portfolio. The average cost of generation by power generation type does not significantly vary across scenarios. The natural gas turbines average levelized cost spans from 242 to 258 $/MWh with the solar scenarios on the lower end and the wind scenarios on the higher end attributable to the start-up costs and lower total production. The most efficient natural gas power plants are dispatched until the cost of carbon is too constraining resulting in the dispatch of hydrogen power plants, first toward the limited capacity fuel cell systems, then toward the hydrogen gas turbine power plants due to high fuel costs.

### Energy storage dispatch

**Figure 5:**
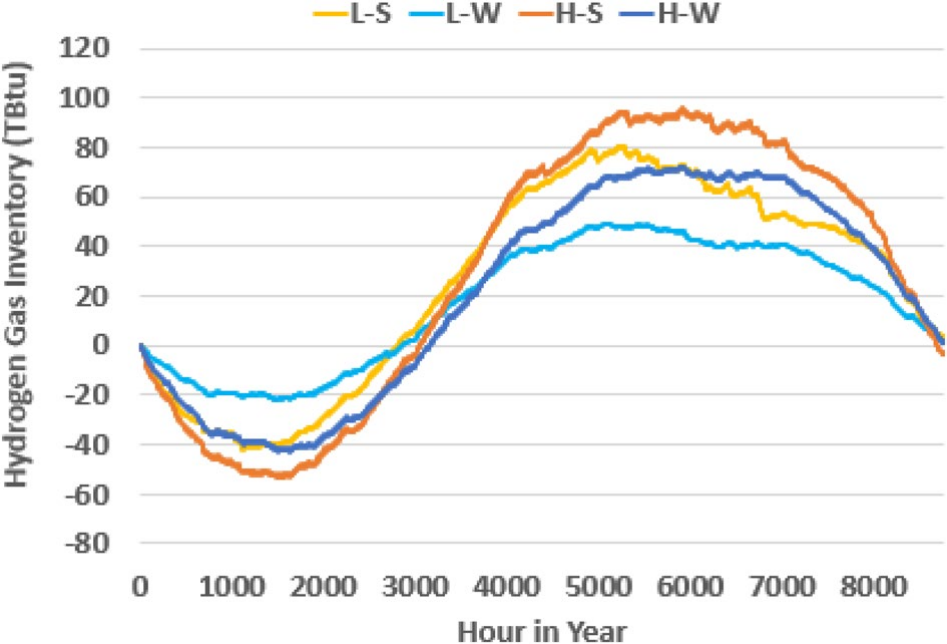
Annual hydrogen gas inventory. Storage amount throughout the simulated year with an arbitrary starting volume and unconstrained storage limits.

In all cases, hydrogen energy storage is adopted to significantly contribute to seasonal energy storage for the electric sector, with hydrogen storage system dynamics for all four scenarios presented in Fig. 5. The resulting required hydrogen storage capacities for the L–S, L–W, H–S, and H–W scenarios are 123, 72, 149, and 115 TBtu, respectively. This can be seen visually in Fig. 5 by evaluating the difference between the minimum and maximum values throughout the year per series. For reference, the working natural gas storage capacity from depleted oil and gas fields in California in 2021 is 339 TBtu^23^, although the volumetric energy density of natural gas is three times that of hydrogen. With the decreased dependence on natural gas, these storage sites may be able to be repurposed for hydrogen storage, the future buildout of a hydrogen infrastructure may also provide an opportunity for massive energy storage via pipeline linepack, and storage assets may be able to be contracted with agencies outside of California. The total storage capacity necessary in wind scenarios is lower due to two factors: (1) the seasonality of hydrogen usage for power generation, but also (2) the dependence on solar availability for hydrogen production. During the winter months, hydrogen consumption in the solar scenarios is higher than in the wind scenarios due to the larger seasonal dependence of solar compared to wind. The sum of the 6 months of the year in which hydrogen is utilized the most for power generation amounts to 28 TWh and 24 TWh of the 36 TWh and 38 TWh annual amounts, or 77% and 73% of the yearly amount, in the L–S and H–S scenarios, respectively. This figure is 11 TWh and 10 TWh, or 71% and 70% of the yearly amount, in the L-W and H-W scenarios, respectively. On the production side, the sum of the best six months of the year in which hydrogen is produced the most uses 174 TWh and 316 TWh of electricity, or 69% and 66% of the annual amount used for hydrogen production, in the L–S and H–S scenarios, respectively. This figure is 117 TWh and 270 TWh, or 72% and 67% of the annual amount used for hydrogen production, in the L–W and H–W scenarios, respectively. In addition, due to the assumption of static hydrogen demand outside of power generation, the true seasonal dynamics of these applications could occur either in synchrony, increasing the storage capacity requirement, or in asynchrony, resulting in a reduction in required storage capacity.

**Figure 6:**
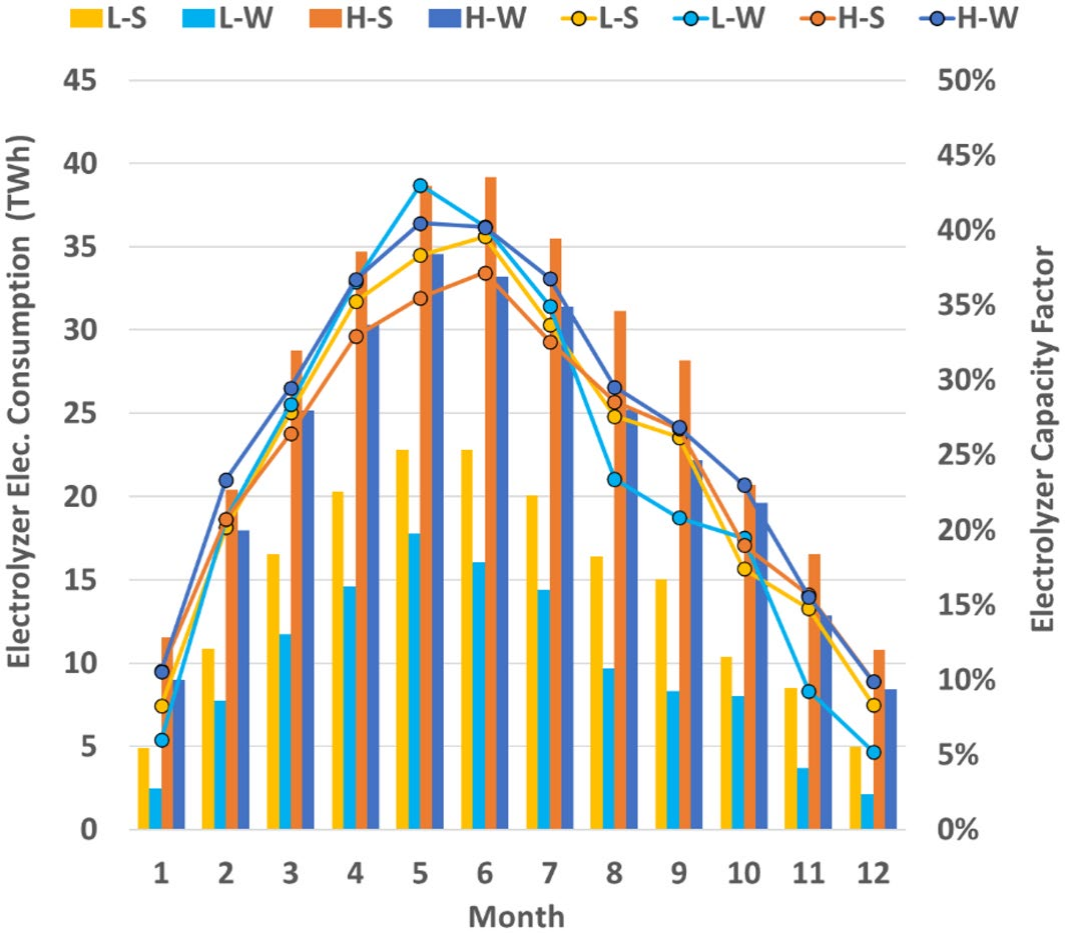
Electrolyzer power consumption and capacity factor. Combined bar and point graph representing monthly electrolyzer electricity consumption and capacity factor, respectively, for the four scenarios.

The differences between electrolyzer capacity factors are within a couple percentage points for all months and scenarios as shown in Fig. 6. The annual electrolyzer CF for L–S and L–W scenarios are both 25%, whereas, for the H–S and H–W scenarios, these numbers are 24% and 27%, respectively. Even when more wind is adopted, excess generation primarily occurs during the hours of solar power generation. Note that the electrolyzer CF between the solar and wind cases is similar because additional solar capacity is deployed proportionally to complement additional electrolyzer capacity. The primary difference between the solar and wind scenarios is the hydrogen demand necessary for power generation, as the solar cases require more hydrogen for fuel to meet nighttime loads. Much more wind capacity than considered here would be required to have wind power surplus during late night toward morning hours. Even then, the higher LCOE of offshore wind may be undesirable as it may be unfavorable relative to much cheaper solar electricity. For this reason, the majority of electrolyzer projects would likely operate close to a solar power plant’s CF, although necessarily lower due to the solar power plant meeting electric load outside of peak hydrogen production hours. Hydrogen production varies in each month throughout the year and total annual hydrogen production varies amongst the scenarios considered (Fig. 6). However, the trend of hydrogen production is generally similar across the scenarios: most of the hydrogen production occurs in the spring and early summer months when solar resource availability is high and coincides with the relatively lower retail electric load. Later in the summer, while the solar resource remains highly available, electricity demands increase due to air conditioning loads.

**Figure 7:**
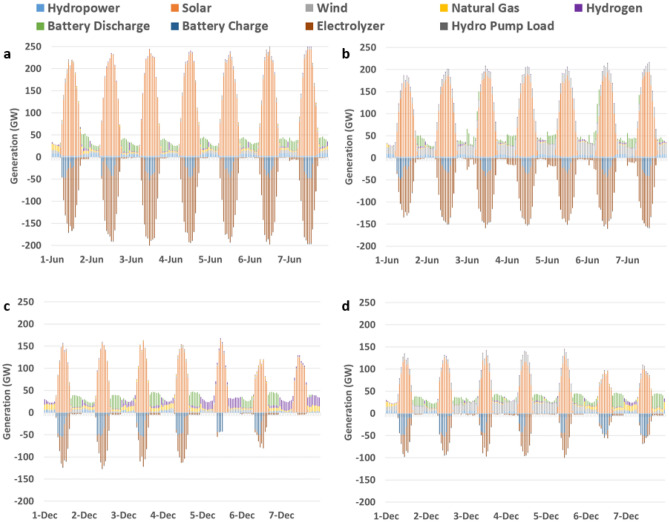
Weekly power generation seasonal snapshots. The first week in (**a**) June H–S scenario, (**b**) June H–W scenario, (**c**) December H–S scenario and (**d**) December H–W scenario.

Curtailment of renewable sources is less than 1% for all scenarios. While this is relatively low compared to the CAISO reported 4% in 2022^44^, this is due to the modeled penalty price for curtailment that would otherwise require even more renewable generation capacity to meet electrolytic hydrogen fuel demand. Balancing this soft constraint to reflect the opportunity cost of developers including the price of renewable fuel credits as explored in Thai and Brouwer^45^ and capture its effect in system level simulations is recommended for future work. The overall capacity of electrolyzers is higher than BESS, which is why across all scenarios more electricity goes toward electrolyzer systems than toward BESS. The amount of electricity going to BESS is 92, 70, 93, and 71 TWh for the L–S, L–W, H–S, and H–W scenarios, respectively. The amount of storage load going to electrolyzer systems is 174, 117, 316, and 270 TWh for the L–S, L–W, H–S, and H–W scenarios, respectively. This goes toward meeting 427, 232, 515, 358 TBtu of hydrogen gas demand for the power generation sector for the L–S, L–W, H–S, and H–W scenarios, respectively. For clarity, Table 3 tabulates these values for a side-by-side comparison. Figure 7 illustrates the large magnitude of solar and loading of electrolyzer systems and BESS in the first week of a Summer and Winter month for the H-demand scenarios.

**Table 3:** Annual hydrogen gas demand by scenario.

Scenario	Non-power generation hydrogen demand (TBtu)	Power generation hydrogen demand (TBtu)
L–S	167	427
L–W	167	232
H–S	563	515
H–W	563	358

**Figure 8:**
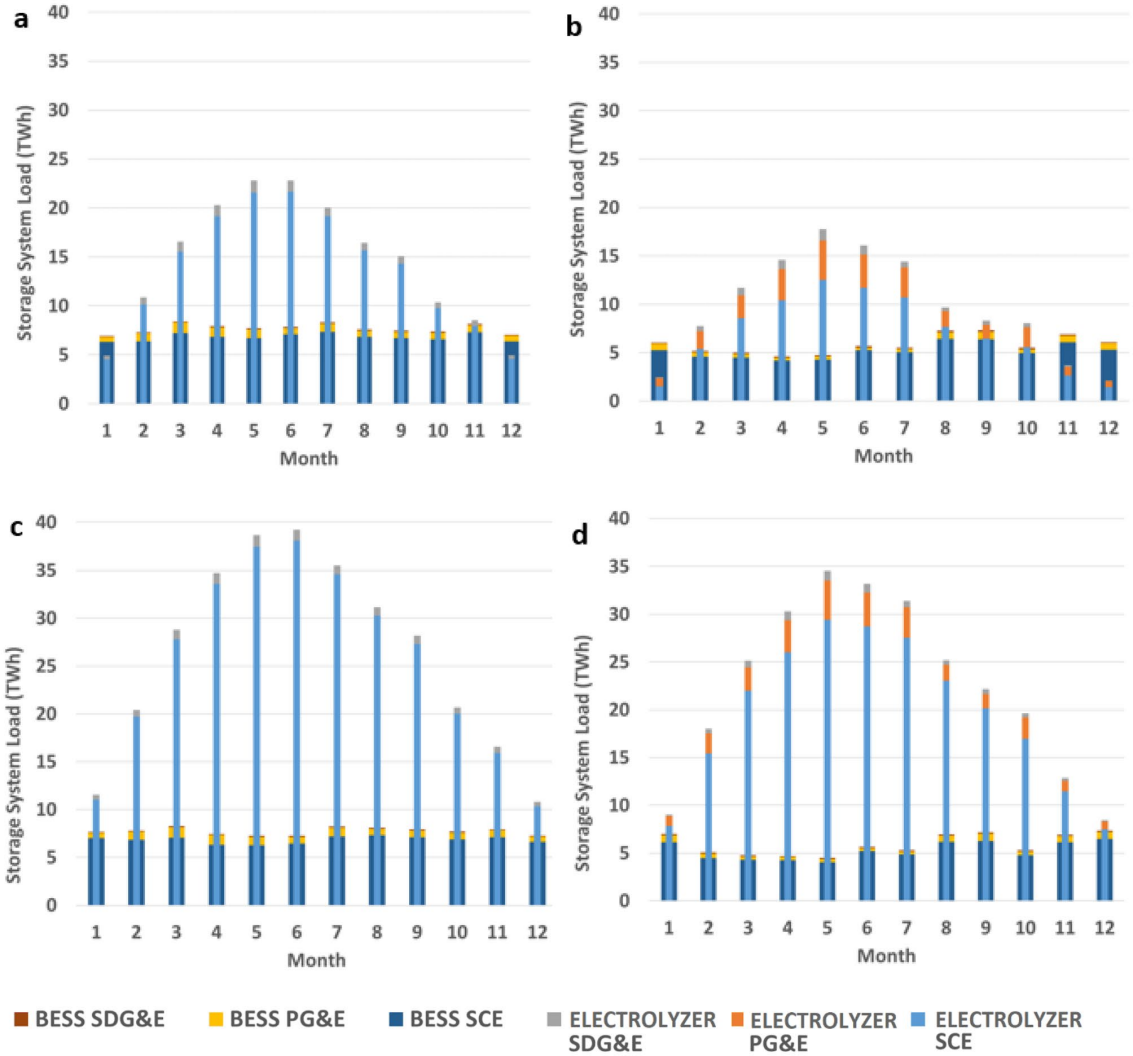
Monthly regional BESS and electrolyzer system electricity usage. (**a**) L–S scenario, (**b**) L–W scenario, (**c**) H–S scenario, and (**d**) H–W scenario.

In terms of storage system loading, by region, SCE service territory generally meets most of the load for both types of storage as this is where most of the utility-scale solar is located. This balance is only shifted in the wind scenarios as all the offshore wind and complementary electrolyzer systems are sited in the PG&E service territory nodes. However, depending on the actual development of offshore wind, the interconnection into the existing electric transmission grid is of key interest to help reduce this discrepancy. Figure 8 portrays the seasonal loading of the storage systems. The amount of electricity sent to SCE and SDG&E BESS are relatively static throughout the year, whereas the PG&E BESS in the wind scenarios have increased seasonal system loading coinciding with increased generation from offshore wind. While this represents a slight seasonal shift in BESS dispatch, all scenarios show that hydrogen energy storage is the primary resource selected for seasonal storage in the electric sector.

Peak hydrogen production occurs in May and June for all scenarios coinciding with the lowest months of electricity going toward BESS by a slight margin in the solar areas. While this might be counterintuitive, as one might expect the increase in solar generation to also result in an increased utilization of BESS, this is primarily due to multiple factors: (1) relatively lower need for BESS to meet night loads in the offshore wind scenarios, (2) relatively low retail electric load in the day coinciding with (3) increased solar generation in the summer evenings. This seasonal mismatch of supply and demand is the same reason driving high solar curtailment in the spring and early summer months of the present-day California grid.

### Regional analysis

Regarding electricity exchange via electric transmission and considering transmission constraints, 46% of imports are purchased from OOS in solar cases and 43% in the wind scenarios. This is a relatively high percentage relative to the approximately 20% as reported by CAISO for 2022^44^. While the adoption of renewable capacity outside of California is uncertain, higher adoption would result in more hours of excess generation that could be sunk in California. The total amount of exports to OOS is only 3% and 2% in the L–S and H–S scenarios, respectively. This number is 1% in both wind scenarios, suggesting load is sufficiently met without regular excess generation available for trading to other balancing authorities outside of the state. The balance of these percentages is electricity exchange between regions as summarized in Supplementary Table 2.

With the majority of the solar sited in the SCE nodes, PG&E relies heavily on imports but less so in the wind scenarios where offshore wind can meet a large portion of load. However, in the solar scenarios a significant portion of solar is sited in SCE territory and exporting out of the region is limited by transmission line capacity. The line connecting SCE territory and PG&E territory as well as the line connecting PG&E valley region to PG&E bay region are fully loaded in more than 60% of the hours throughout the year in the solar scenarios, occurring mostly during solar generation timeframes. These two lines are similarly loaded as there is no direct connection between SCE territory and PG&E bay area with the latter being a major load center. The number of congested hours drops to roughly a third of all annual hours in wind scenarios (see Supplementary Fig. 2).

The loading duration curves of the transmission lines between SCE territory nodes do not change too significantly between scenarios. Only the line connecting the eastern area to the remainder of the SCE territory is congested in the summer months during peak solar generation as the node with the highest share of solar generation capacity. All the other SCE nodes generally are not congested due to the amount of energy storage available to act as a sink for excess generation. A notable result is that congestion occurs the most in the evening when the BESS that are co-located at the PV plants must dispatch to meet local loads and transmit electricity to meet the major evening load of the metropolitan area within SCE territory. This also occurs to a lesser degree on the line connecting the PG&E valley node with the PG&E bay area node, most prominently seen in the evening hours from February to June in the solar scenarios. Tables presenting the average remaining overhead capacity of each line by month and hour to quickly identify these trends are available in Supplementary Information.

## Discussion

If the development of offshore wind capacity is possible, it would generally result in better system performance in terms of required transmission capacity, cost, and emissions up to the point where nighttime until dawn loads are mostly met. Because LCOH is sensitive to the cost of feedstock electricity, low-cost solar still seems to be the best source for electrolytic hydrogen production, effectively providing a ceiling for electrolyzer CF. Any solar capacity deployed that is not already mostly meeting electric loads or charging BESS for evening dispatch would not be deployed at all if not for the purpose of renewable fuel production. The investigated amount of renewable hydrogen fuel demands considered resulted in significant loading of electrolyzer systems amounting to 21% to 41% of total electric load in the scenarios considered.

Fuel production and consumption are not as time constraining as meeting electric loads (even BEV charging) so that this cross-sectoral type of scenario provides a strong opportunity to evaluate the value of flexible GW-scale electrolyzer systems and how these can balance load-supply mismatch in a deep renewable power generation sector. At the same time, these large-scale electrolytics hydrogen production capabilities provide seasonal storage of grid electricity and transportation electrification attributes. Due to the magnitude of the formulation in this work, only one weather year was considered. However, the insights generated from this work can provide guidelines for future works to evaluate the annual variability in seasonality and consequently the assets required. In addition, seasonal demand for hydrogen outside of the power generation sector could be accounted for.

The exception to the electrolyzer CF ceiling would be the availability of onshore wind generation and lifting the limitation on OOS imports to supplement local solar power generation. This is important because reducing the cost of hydrogen consequently affects the opportunity to dispatch hydrogen powered fuel cells, promoting capacity deployment to meet load that would otherwise be met by natural gas power plants. However, due to the nature of bulk energy transmission it would be more likely for OOS to produce and import hydrogen than to build additional electric transmission capacity to deliver feedstock electricity specifically for hydrogen production. To this end, the key assumptions that are worth re-evaluating in future work from this study would be the assumption that (1) 10% CF and the resulting cash flow is the marginal economically viable deployment, as opposed to higher or lower and (2) the availability of hydrogen imports from OOS.

Transmission between inter-state regions is fairly congested with most of the challenge arising from meeting evening and late-night loads. If the solution for this is not to increase transmission line capacity to deliver PV + BESS electricity, it may be the usage of hydrogen pipelines for fuel cell power systems closer to load centers. In this study, fuel cell capacities were distributed proportionally to utility-scale solar and BESS deployment, however it may be possible to skew deployment towards major load centers to circumvent electric transmission. Future studies that may investigate the sensitivities of the LCOH may also want to account for the value of being able to defer electric transmission upgrades in favor of lower cost hydrogen pipelines as explored in Thai and Brouwer^26^.

Increased electrolytic hydrogen production for applications outside of the power generation sector reduces carbon emissions but results in an order of magnitude smaller increase in carbon emissions within the power generation sector due to the shrinking opportunity for fuel cell systems to operate economically with a higher level of solar generation. If there is not a surplus of renewable generation from midnight to dawn either due to increased future overnight loads or lack of renewable capacity, electrolyzer systems will be operated to strongly follow PV generation profiles, which produces a relatively low ceiling on CF at a value of roughly 25%. Despite this, the LCOH is more sensitive to the price of feedstock electricity than it is to the capital cost or CF of the electrolyzer system. This in turn affects the level of dispatch of fuel cell power systems using hydrogen and the remaining flexible needs that are met by natural gas power plants driving up power generation sector costs.

Massive amounts of gas storage are required for hydrogen for seasonal storage amounting to between 72 and 149 TBtu for the scenarios considered. Hydrogen gas storage requirement and consumption is lower in the wind scenarios due to complementary seasonal generation of solar and offshore wind but also because wind can meet more nighttime loads reducing hydrogen consumption for power generation outside of solar generation hours. BESS operation in the solar scenarios is relatively static throughout the year but is more active in winter months when significant wind capacity is deployed. Surprisingly, BESS and electrolyzer electricity consumption is somewhat inversely related, with BESS operating relatively lower in the middle of the year corresponding to peak electrolyzer operation.

Overall, the role of electrolyzers and fuel cells largely complements those that cannot be met through BESS technologies to achieve carbon neutrality. These applications typically end up being seasonal storage and hard-to-decarbonize applications, with one of the largest markets being for freight. While the short-term challenges of decarbonization can be addressed with BESS (e.g., increasing power generation RES%, electrification of passenger transportation, electrification of some heating demands), further looking challenges will require an alternative approach due to self-discharge and the coupled power-energy capacity nature of BESS. Early investment in the hydrogen vector will allow flexibility in decarbonizing various applications with synergistic effects. For example, electrolyzers in both distributed and transmission settings could enable higher levels of local PV generation—increasing electric grid renewable content and injecting renewable hydrogen into the gas grid otherwise. Garnering experience in deploying and integrating hydrogen systems will pay dividends when tackling the hardest to decarbonize applications. Significant investments and policies are required to generate momentum for long-term success.

## Conclusion

To conclude, a spatially-resolved hourly annual statewide power generation system least-cost economic dispatch optimization is carried out for four different scenarios representing two levels of hydrogen demand for applications outside of the power generation sector. Half of the scenarios model 21 GW of offshore wind capacity resulting in lower total generation and cost for each respective level of hydrogen demand. A carbon price of 50 $/tonne price is sufficient to offset the increased cost of hydrogen fuel production and conditioning for transportation applications to be at parity with nonrenewable fuel costs. The total cost of power generation is 8% and 11% higher in the high hydrogen demand scenarios at the benefit of decreasing CO_2_ emissions by 19 MMT in the solar scenario and by 22 MMT in the wind scenario compared to the low demand scenarios, respectively. This is a 45% and 73% carbon emissions reduction from between the low and high hydrogen demand scenarios that utilize less hydrogen equivalent to an emission reduction carbon abatement cost of 45 and 34 $/MTCO_2_e for the solar and wind scenarios, respectively. Despite the relatively higher cost of offshore wind, the overall system cost is roughly 16% lower than a solar dominant portfolio due to favorable dynamics to meet nighttime loads directly. 115 TBtu of seasonal storage is required for a 90% renewable energy supply power generation sector with conservative hydrogen adoption for the transportation sector and could be further refined when considering the seasonality of hydrogen demand outside of the power generation sector.

While the analyses of this work are conducted with sensitivities for many changing variables, the roles that hydrogen would fill in the power generation and transportation sectors are unlikely to change. This study finds that generally more hydrogen adoption enables further decarbonizing the transportation sector and that offshore wind will largely reduce system costs relative to more solar and storage. However, the possibility of this will largely depend on policy which enables further technology maturation and public support. Future work should consider exploring various policies and incentives that would significantly affect the economic viability of deploying hydrogen systems. In addition, interconnected regional analyses are recommended with a focus on hydrogen effectively replacing the existing natural gas grid system between states.

### Supplementary Information

Supplementary Information 1.

Supplementary Table S4.

Supplementary Information 2.

Supplementary Information 3.

## Data Availability

The data that support the analyses within this paper and other findings of this study are available from the corresponding author upon reasonable request. The source data underlying Figs. 1, 2, 3, 4, 5, 6, 7 and 8 is provided as a Source Data file.
