# Supplementary material for: Formal optimization techniques select hydrogen to decarbonize California
Source: Sci Rep. 2024 Jan 29;14:2435. doi: 10.1038/s41598-024-52157-6 (PMC10825219; doi:10.1038/s41598-024-52157-6)
Supplement: Supplementary file 3 — Supplementary Information 2. [file 41598_2024_52157_MOESM3_ESM.pdf]

Supplementary Tables

Supplementary Table 1 – Capacity factor of generators by fuel type.

	L-S	L-W	H-S	H-W
**Nuclear**	85%	85%	85%	85%
**Geothermal**	80%	80%	80%	80%
**Solar**	25%	25%	26%	25%
**Wind**	21%	45%	21%	45%
**Hydropower**	28%	28%	29%	29%
**Natural Gas**	15%	5%	15%	5%
**Biomass**	85%	83%	85%	83%
**Hydrogen (GT)**	50%	48%	50%	49%
**Hydrogen (FC)**	11%	10%	11%	8%
**BESS**	14%	10%	14%	10%

Supplementary Table 2 – Electricity exchange for each IOU regions by scenario.

Scenario	Region	Import (TWh)	Export (TWh)
L-S	PG&E	105	30
	SCE	34	31
	SDGE	19	5
L-W	PG&E	56	36
	SCE	60	10
	SDGE	22	10
H-S	PG&E	105	30
	SCE	34	30
	SDGE	19	5
H-W	PG&E	57	35
	SCE	57	11
	SDGE	22	9

Supplementary Table 3 – Electric transmission line flow capacities

From Node	To Node	Maximum Flow (MW)	Minimum Flow (MW)
CIPB	CIPV	3500	-3500
CIPV	CISC-TabCC	4000	-3000
CISC-Metro	CISD	4100	-2500
CISC-East	CISC	2000	-2000
CISC-EoL	CISC	5000	-5000
CISC-Metro	CISC	14500	-14500
CISC-NoL	CISC	1500	-1500
CISC-TabCC	CISC	6000	-6000
External	CISC	13500	-12500
External	CISD	4200	-3800
External	CIPV	7800	-6600

Supplementary Figures

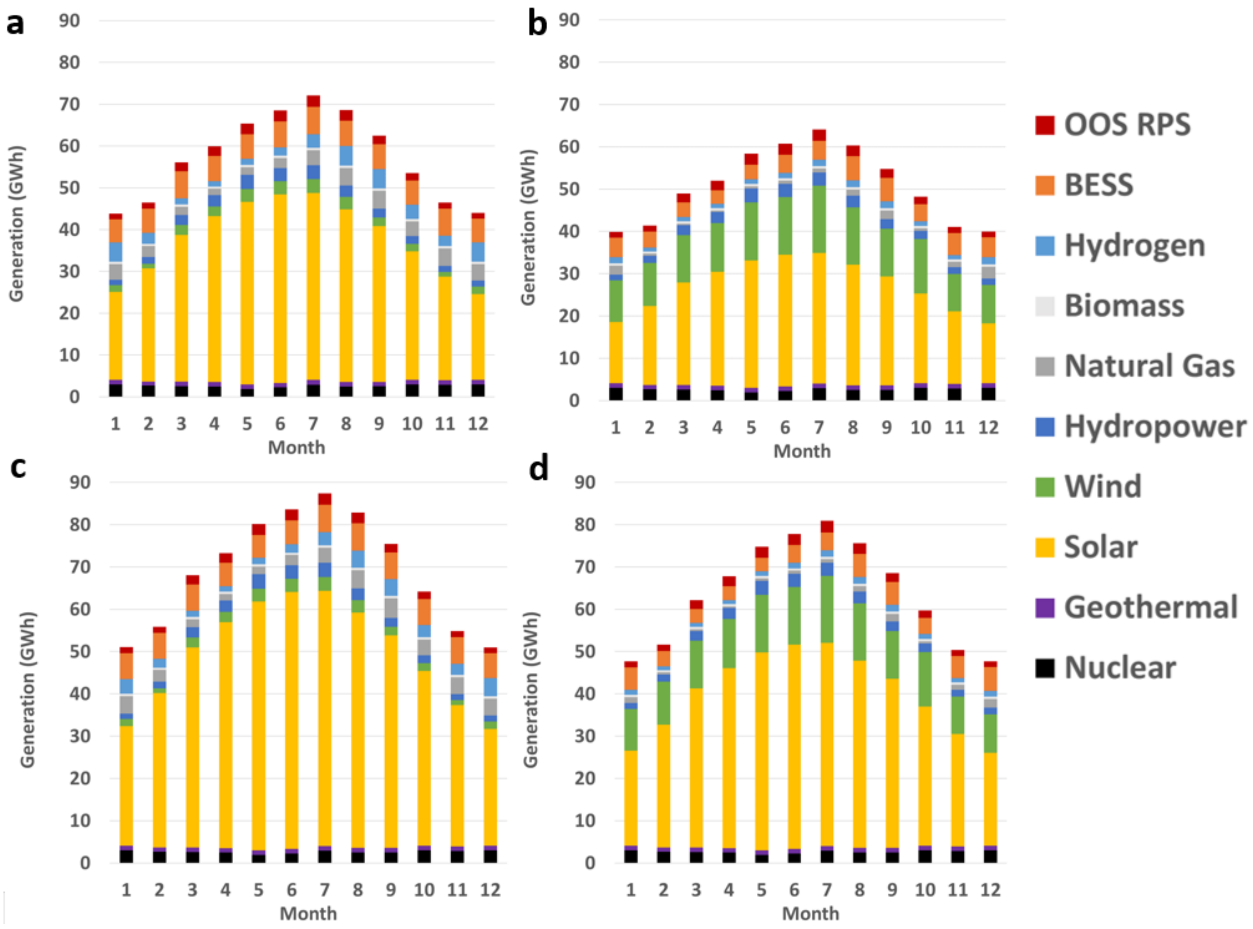

Supplementary Figure 1 – Monthly electricity generation by fuel type for a) L-S scenario, b) L-W scenario, c) H-S scenario, and d) H-W scenario.

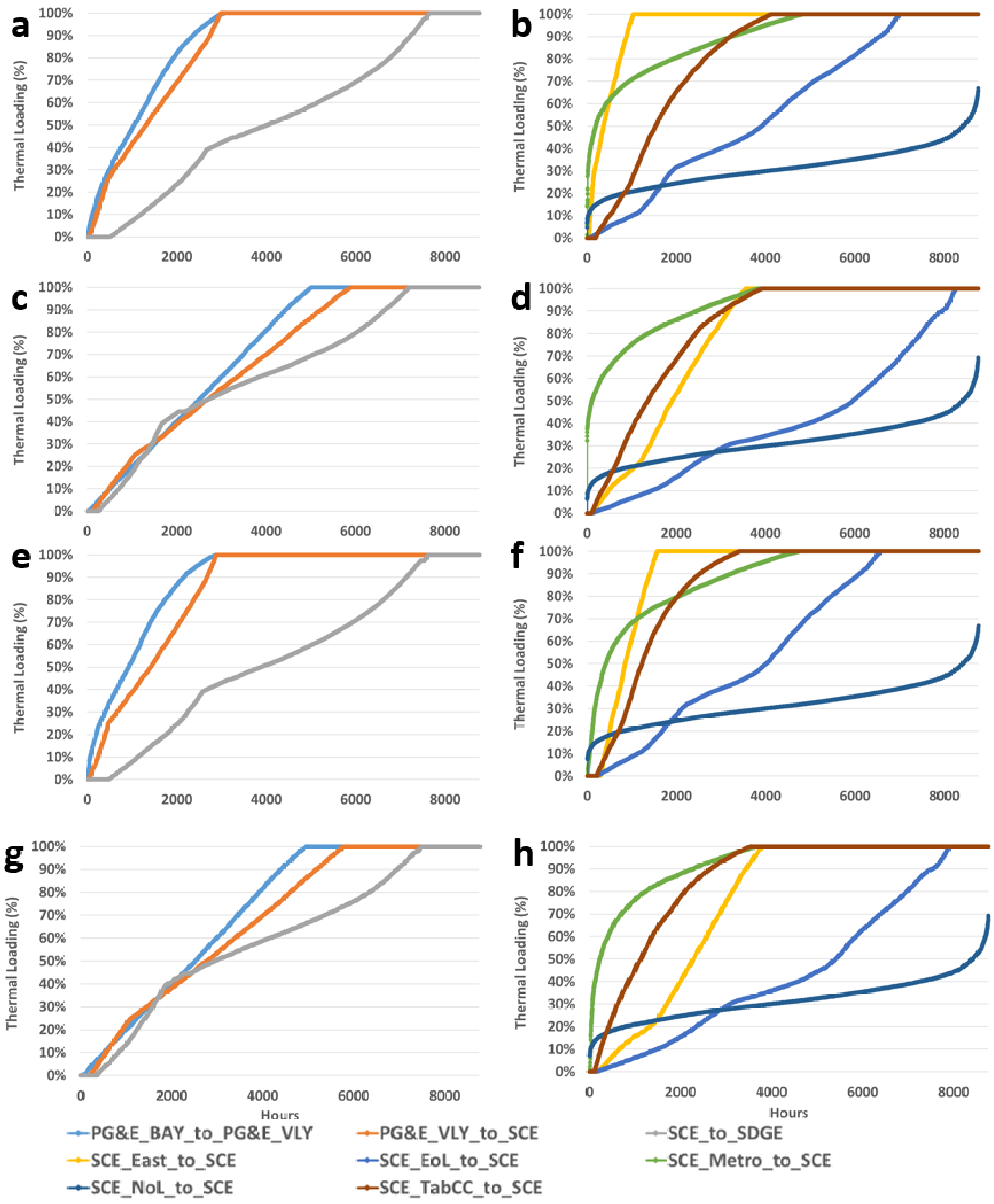

Supplementary Figure 2 – Thermal loading duration curve for transmission lines a) between L-S scenario IOU regions b) within L-S scenario SCE region c) between L-W scenario IOU regions d) within L-W scenario SCE region e) between H-S scenario IOU regions f) within H-S scenario SCE region g) between H-W scenario IOU regions h) within H-W scenario SCE region
